# Getting Cartilage Thickness Measurements Right: A Systematic Inter-Method Comparison Using MRI Data from the Osteoarthritis Initiative

**DOI:** 10.1177/19476035221144744

**Published:** 2023-01-19

**Authors:** Teresa Nolte, Simon Westfechtel, Justus Schock, Matthias Knobe, Torsten Pastor, Elisabeth Pfaehler, Christiane Kuhl, Daniel Truhn, Sven Nebelung

**Affiliations:** 1 Department of Diagnostic and Interventional Radiology, University Hospital Aachen, Aachen, Germany; 2 Faculty of Medicine, University of Zurich, Zurich, Switzerland; 3 Faculty of Medicine, University Hospital Aachen, Aachen, Germany; 4 Department of Orthopaedic and Trauma Surgery, Lucerne Cantonal Hospital, Lucerne, Switzerland; 5 Institute for Advanced Simulation, Forschungszentrum Jülich, Jülich, Germany

**Keywords:** cartilage thickness, inter-method comparison, osteoarthritis, magnetic resonance imaging, osteoarthritis initiative

## Abstract

**Objective:**

Magnetic resonance imaging is the standard imaging modality to assess articular cartilage. As the imaging surrogate of degenerative joint disease, cartilage thickness is commonly quantified after tissue segmentation. In lack of a standard method, this study systematically compared five methods for automatic cartilage thickness measurements across the knee joint and as a function of region and sub-region: 3D mesh normals (3D-MN), 3D nearest neighbors (3D-NN), 3D ray tracing (3D-RT), 2D centerline normals (2D-CN), and 2D surface normals (2D-SN).

**Design:**

Based on the manually segmented femoral and tibial cartilage of 507 human knee joints, mean cartilage thickness was computed for the entire femorotibial joint, 4 joint regions, and 20 subregions using these methods. Inter-method comparisons of mean cartilage thickness and computation times were performed by one-way analysis of variance (ANOVA), Bland-Altman analyses and Lin’s concordance correlation coefficient (CCC).

**Results:**

Mean inter-method differences in cartilage thickness were significant in nearly all subregions (*P* < 0.001). By trend, mean differences were smallest between 3D-MN and 2D-SN in most (sub)regions, which is also reflected by highest quantitative inter-method agreement and CCCs. 3D-RT was prone to severe overestimation of up to 2.5 mm. 3D-MN, 3D-NN, and 2D-SN required mean processing times of ≤5.3 s per joint and were thus similarly efficient, whereas the time demand of 2D-CN and 3D-RT was much larger at 133 ± 29 and 351 ± 10 s per joint (*P* < 0.001).

**Conclusions:**

In automatic cartilage thickness determination, quantification accuracy and computational burden are largely affected by the underlying method. Mesh and surface normals or nearest neighbor searches should be used because they accurately capture variable geometries while being time-efficient.

## Introduction

Osteoarthritis (OA) leads to the progressive and irreversible loss of articular cartilage in the peripheral and the axial skeleton joints.^1^ OA is highly prevalent and its global burden is increasing, which is aggravated by aging populations and the increasing prevalence of other risk factors.^2,3^

Magnetic resonance imaging (MRI) offers excellent soft-tissue contrast and is considered the clinical standard of reference for the diagnosis of OA and other joint diseases of all joint tissues involved in the disease of OA.^4^ On morphologic MR images, the structural degeneration and ultimate loss of cartilage translate to locally reduced cartilage thickness.^5,6^

Manually measuring cartilage thickness across the knee joint, however, remains a time-consuming task.^7^ The problem can be addressed by fully automated approaches, which typically require segmenting cartilage in the MR images, defining joint regions and subregions, and calculating mean cartilage thickness per region and subregion. Different approaches for cartilage thickness measurements have been developed previously, for example, (1) finding the minimum distance between opposite cartilage surfaces,^8-12^ (2) measuring the lengths of surface normals between 3D meshes that approximate the cartilage surfaces,^6,8,11,13^ (3) combining surface normals with offset maps,^14^ (4) analyzing shape models fitted to the cartilage volume,^15^ or (5) computing curved potential field lines between the cartilage surfaces.^11^ Commercially available techniques also rely on the calculation of surface normals between meshes,^6^ which may thus be considered the most established method to date.

Against this background, the present study aimed to systematically compare a variety of established and new methods to determine cartilage thickness as a function of joint region and subregion. Beyond considering agreement between the methods, we also aimed to assess the computational burden of each method and, thus, their suitability for larger-scale analyses. Our hypothesis was that the distinctly different methodologic approaches are characterized by variable cartilage thickness measurements, inter-method agreements, and associated computational burden.

## Method

### Dataset

The data of this study, that is, MRI exams of the knee joint, were provided by the Osteoarthritis Initiative (OAI), which is a prospective, multi-center, longitudinal, and observational clinical trial that studies the progression of knee OA.^16^ For the baseline measurements, 507 manually segmented outlines of femorotibial cartilage have recently been published as the OAI-ZIB (Zuse Institute Berlin) dataset,^17^ and these data were used to develop and implement the methods of the present study.^18^ The MRI scans had been acquired at baseline using a double echo steady state (DESS) sequence run on a 3T clinical MRI scanner (Siemens Trio, Erlangen, Germany), a resolution of 0.36 x 0.36 x 0.7 mm³, and sagittally oriented slices. The study cohort consisted of 262 male and 245 female study participants with a mean age of 61.87 ± 9.33 years and a mean Body Mass Index of (29.27 ± 4.52) kg/m². In all, 60, 77, 61, 151, and 158 study participants presented with radiographic OA grades of 0, 1, 2, 3, and 4, respectively, thereby displaying a moderate bias toward more severe radiographic OA grades. Radiographic OA grades are defined by the OAI as “quasi Kellgren Lawrence grades.”^17,19^ Through their respective labels, each voxel in the three-dimensional scan matrix had been allocated to different regions such as the tibial or femoral cartilage via an integer encoding.

### Data Preprocessing

**Figure 1.**
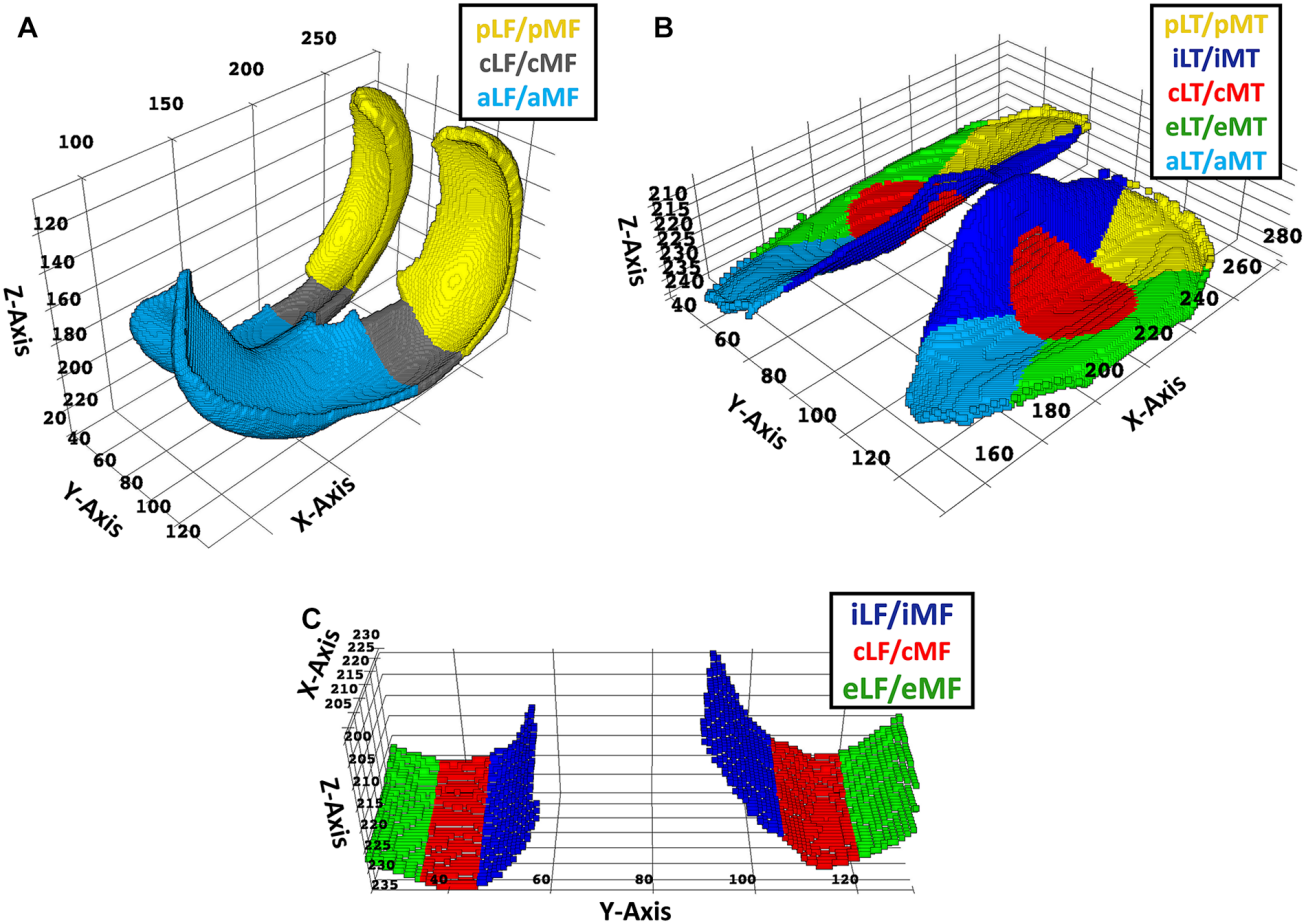
Visualization of the femoral and tibial cartilage subregions. Based on manually segmented cartilage volumes, the femoral cartilage subregions (**A**), a close-up of the central femoral subregions (**B**), and the tibial cartilage subregions (**C**) are shown. Subregions were defined in line with Wirth and Eckstein^23^ as three femoral and five tibial subregions per compartment, that is, anterior [a], central [c], and posterior [p] for the femur, and -additionally- internal [i] and external [e] for the tibia. The central, that is, weight-bearing, femoral subregions were defined as being vis-à-vis the central tibial subregions and were subdivided further into the internal central (icLF, icMF), central central (ccLF, ccMF), and external central subregions (ecLF, ecMF) as shown in (**B**). For the coordinate system, the x-axis was defined along the anteroposterior dimension, the y-axis along the mediolateral dimension, and the z-axis along the head-feet direction. Acronyms designating the subregions are summarized in **Table 1**. Units of axes are voxels.

The segmented knee joints were first converted from MHD (MetaImage Header) format to numpy arrays using the SimpleITK library.^20^-^22^ All subsequent data processing steps were implemented in Python (version 3.9.5). The segmented voxels of the tibial and femoral cartilage were extracted based on their assigned integer values in the source-segmentation files. Thereby, two distinct point clouds representing the tibial and femoral segmentation volumes were obtained and subsequently divided into distinct anatomic subregions following the approach of Wirth and Eckstein^23^ and using customized Python routines. More specifically, the tibial segmentation volume was split into medial and lateral along the central y-coordinate. Both cartilage plates (CPs) were then divided into a central subregion (central medial tibia [cMT], central lateral tibia [cLT]) and an outer region. To this end, a cylinder was placed around the center of gravity of the respective CP with its axis in z-direction and dimensioned to account for 20% of the plate volume. The outer region around the cylinder was further divided into four separate subregions, that is, the anterior (anterior medial tibia [aMT], anterior lateral tibia [aLT]), the posterior (posterior medial tibia [pMT], posterior lateral tibia [pLT]), the internal (internal medial tibia [iMT], internal lateral tibia [iLT]), and the external (external medial tibia [eMT], external lateral tibia [eLT]) subregions.^23^ The femoral segmentation volume was split into medial and lateral along the trochlear sulcus. Both CPs were then divided along the x-axis into anterior subregions (anterior medial femur [aMF], anterior lateral femur [aLF]), central subregions (central medial femur [cMF], central lateral femur [cLF]), and posterior subregions (posterior medial femur [pMF], posterior lateral femur [pLF]). The central femoral subregions were defined as being vis-à-vis the corresponding central tibial subregions, with the anterior and posterior subregions being adjacent to the central femoral subregions. The central femoral subregions were further subdivided along the y-axis into internal (internal central medial femur [icMF], internal central lateral femur [icLF]), central (central central medial femur [ccMF], central central lateral femur [ccLF]), and external (external central medial femur [ecMF], and external central lateral femur [ecLF]) subregions, which comprised a third of the central femoral subregion each. **Figure 1** visualizes the different cartilage subregions along the Cartesian coordinate system, while **Table 1** summarizes the cartilage subregions and their acronyms. In addition, we also considered the entire cartilage of a joint, that is, globally, and the regions, that is, the medial and lateral tibia (MT, LT) and the medial and lateral femur (MF, LF), respectively.

**Table 1.** Nomenclature of Femoral and Tibial Subregions and Their Acronyms as Standardized by Wirth and Eckstein.^23^

Acronym	Meaning
Tibia	
cLT/cMT	Central subregion of the lateral/medial tibia
eLT/eMT	External subregion of the lateral/medial tibia
iLT/iMT	Internal subregion of the lateral/medial tibia
aLT/aMT	Anterior subregion of the lateral/medial tibia
pLT/pMT	Posterior subregion of the lateral/medial tibia
Femur	
cLF/cMF	Central subregion of the lateral/medial femur
ccLF/ccMF	Central subregion of the clf/cmf (“central central”)
ecLF/ecMF	External subregion of the clf/cmf (“external central”)
icLF/icMF	Internal subregion of the clf/cmf (“internal central”)
aLF/aMF	Anterior subregion of the lateral/medial femur
pLF/pMF	Posterior subregion of the lateral/medial femur

In contrast to their approach, we also analyzed the anterior and posterior femoral subregions.

### Methods for Cartilage Thickness Measurement

**Figure 2.**
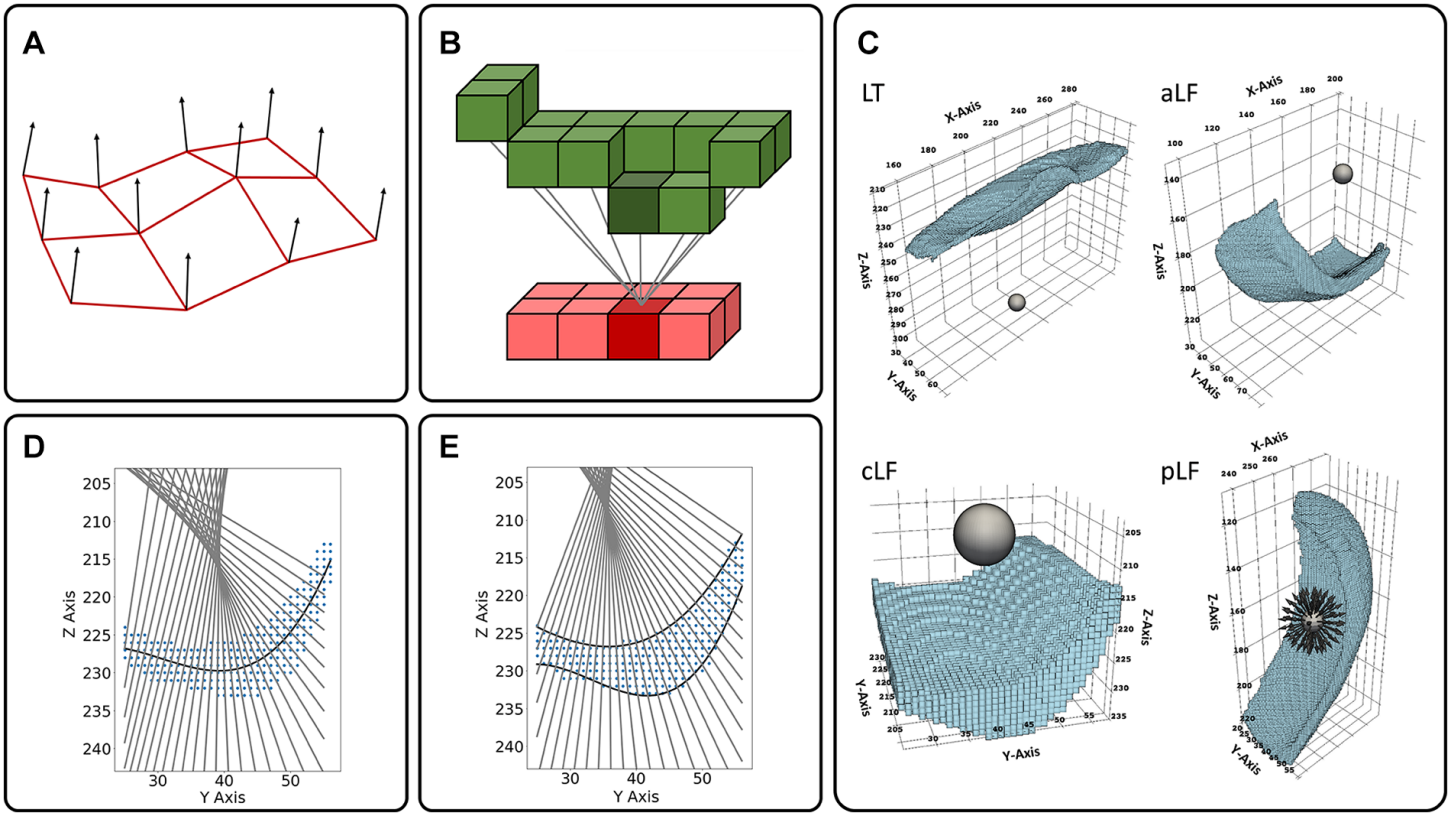
Schematic visualizations of the methods for automated cartilage thickness measurements. (**A**) 3D mesh normals (3D-MN): Schematic representation of distal surface meshes (red) and normals at their vertices (black arrows). (**B**) 3D nearest neighbors (3D-NN): For every voxel of the distal cartilage surface (red), the corresponding voxel of the proximal cartilage surface with the minimum Euclidian distance is determined (green). (**C**) 3D ray tracing (3D-RT): The positions of the spheres that were defined as the origins of the bundle of rays are shown for the lateral tibia (LT) and for the anterior (aLF), central (cLF), and posterior (pLF) lateral femur. Separate spheres were positioned for each of these subregions (as well as for their medial counterparts [not shown]). For the pLF, schematic rays originating from the sphere are visualized. (**D**) 2D centerline normals (2D-CN): Through the segmented cartilage area (blue), the polynomial fit function was determined to approximate the centerline (black). Normals to the centerline (gray) were used for thickness calculation. Example slice through the tibial cartilage. (**E**) 2D surface normals (2D-SN): The cartilage surfaces were fit using polynomial functions (black) and used to define normals to the distal fit functions (gray). Color-coding and tibial slice as in (**D**). Units of axes are voxels.

Altogether, five different methods to automatically determine tibial and femoral cartilage thickness were implemented and comparatively evaluated (**Fig. 2**). While three methods (i.e., 3D mesh normal [3D-MN], 3D nearest neighbors [3D-NN], and 3D ray tracing [3D-RT]) function in the 3D space, the two other methods (i.e., 2D centerline normals [2D-CN] and 2D surface normals [2D-SN]) use 2D sagittal images and were, thus, applied slice by slice. Importantly, all methods consider the full extent of the segmented cartilage. In the following, we will refer to the cartilage surfaces as “distal” or “proximal.” For the tibia, the distal cartilage surface follows the cartilage-bone transition, while the proximal cartilage surface follows the cartilage-synovia transition. For the femur, this is vice-versa.

#### 3D mesh normals (3D-MN)

To implement the 3D-MN method, we defined normals along the meshed distal cartilage surface and tracked their intersection with the proximal cartilage surface mesh (**Fig. 2A**). First, the surface voxels of the tibial and the femoral segmentation volumes were extracted to function as mesh vertices. For the tibia, the voxel with the highest z-value for a given (x, y) coordinate was assigned to be a vertex of the distal mesh, while, correspondingly, the voxel with the lowest z-axis value was assigned to be a vertex of the proximal mesh. For the femoral segmentation volume, this approach needed to be modified because lines along the z-axis regularly intersected the curved segmentation volume of the posterior subregions twice. Hence, the posterior subregions were rotated by 90° around the y-axis to be parallel with the x-y-plane before the surface voxels were extracted and assigned to be vertices of the proximal and distal mesh of the femur. Second, meshes were constructed by Delaunay triangulation^24^ and one surface normal vector was subsequently calculated per mesh vertex of the distal tibial and femoral meshes, respectively. Here, a surface normal vector was defined as the vector that was perpendicular to the tangent plane of the mesh surface at the respective vertex. Averaged over all 507 knee joints, one mesh element had an area of 0.19 and 0.27 mm² for the tibia and the femur, respectively. Third, cartilage thickness was measured by tracing the lengths of the normal vectors from their origin at the distal mesh to the intersection with the respective proximal mesh using the ray_trace function from the pyvista library.^25^ Cartilage thickness values were allocated to the joint’s subregions based on the (x, y,z) coordinates of the distal mesh vertices. Please note that alternative 3D-MN implementations as, for example, by Wirth and Eckstein^23^ weighted the individual thickness measurements by the mesh element size and used bi-directional surface normals from distal to proximal and vice versa.

#### 3D nearest neighbors (3D-NN)

To implement the 3D-NN method, we performed distance measurements between voxels from the distal cartilage surface and their nearest neighboring voxels from the proximal cartilage surface (**Fig. 2B**). Similar to the 3D-MN method, the distal and proximal surface voxels of the tibial and femoral segmentation volume were extracted. For every voxel along the distal surface, the nearest neighboring voxel along the proximal surface was determined by a nearest neighbor search over all proximal surface voxels, making use of a 3-dimensional search tree structure (KDTree) from the scipy library.^26^ Cartilage thickness was measured as the Euclidian distance to the resulting nearest neighbor and individual values were assigned to the joint’s subregions based on the (x, y,z) coordinate of the distal surface voxel.

#### 3D ray tracing (3D-RT)

To implement the 3D-RT method, we determined the points of intersection of a bundle of rays with the cartilage surfaces (**Fig. 2C**). For the tibia (and femur), two (and six, respectively) spheres were positioned in proximity of the cartilage within the respective bone. More specifically, one sphere each was used for the LT, MT, aLF, aMF, cLF, cMF, pLF, and pMF and positioned centrally below the LT and MT or near the focal point of the curved aMF, aLF, cMF, cLF, pLF and pLM, respectively. For further details please refer to Appendix. By attributing 60 surface vertices along the polar direction and 60 surface vertices along the azimuthal direction, 3482 evenly distributed normal vectors or “rays” were defined per sphere using the sphere function from the pyvista library.^25^ Originating at the vertices of the respective spheres as defined above, each ray was extended to enter and leave the segmented (sub)region at two distinct points of intersection. Cartilage thickness was determined as the Euclidian distance between these two points. For rays that did not intersect with the segmented volume after a maximum of 100 iterations, no thickness value was calculated. Cartilage thickness values were allocated to the joint’s subregions based on the (x, y,z) coordinates where a specific ray first entered the respective subregion.

#### 2D centerline normals (2D-CN)

To implement the 2D-CN method, we determined normals to fit function that approximates the centerline through the cartilage (**Fig. 2D**). For every sagittal image of the tibial or femoral cartilage, a third-order polynomial function was fit to approximate the centerline of the respective segmented cartilage area. The polynomial degree of three was chosen because, in practice, no more than two inflection points per layer were observed and restraining the polynomial degree to three helped avoid overfitting and oscillations of the fit function at the edges of the cartilage surface (i.e., Runge’s phenomenon)^27^ that might occur with higher polynomial degrees. As the cartilage cross-sectional area extends by $N_{x}$ voxels along the *x*-axis, $N_{x}$ normal vectors to the fit function were calculated per image. One cartilage thickness value per normal vector was then determined by finding the proximal and distal intersection points of the normal vectors with the cartilage outlines and by subsequent computation of the Euclidean distance between both intersection points. Cartilage thickness values were allocated to the joint’s subregions based on the (x, y, z) coordinates of the more distal intersection point.

#### 2D surface normals (2D-SN)

To implement the 2D-SN method, we approximated the distal and proximal cartilage outline by two fit functions and determined the intersection of normals to the distal fit function with the proximal one (**Fig. 2E**). On each sagittal image, two functions $f_{d}$ and $f_{p}$ , where *d* indicates distal and *p* proximal, were fit to the distal and proximal cartilage surfaces per femoral or tibial cartilage outline. For similar reasons as laid out for the 2D-CN method, functions were third-order polynomials. Within a 2D image of the same coordinate y (corresponding to the sagittal view), one cartilage thickness value per voxel along the x-axis was determined by calculating the normal to the distal fit function at the point $\left(x,f_{d}(x)\right)$ and its intersection point with the proximal fit function $f_{p}$ . Cartilage thickness values were allocated to the joint’s subregions based on x , $f_{d}(x)$ and the image coordinate y .

### Performance Analysis

On a per-knee basis, average computation times (as surrogates of the computational burden of each method) were determined by processing a subset of 50 segmented knee joints on a standalone PC with the following specifications: AMD Ryzen 7 3800X 8-Core Processor (3901 MHz, 8 cores, 16 logical processors) and 32 GB RAM. For 3D-MN, 2D-CN, and 2D-SN, 16 segmented knees at a time were processed in parallel. For 3D-RT and 3D-NN, parallelization (using the 16 logical cores) took place inside the methods themselves. Besides computation times, the average number of measurement points per tibial and femoral segmentation volume was determined, that is, how many normals, nearest neighbor searches, or rays, respectively, contributed to cartilage thickness calculation on average.

### Statistical Analysis

Mean cartilage thickness values were determined globally and per region and subregion (as defined in **Table 1**) using the five methods. Outliers were defined on a subregional basis, that is, as those segmented knee joints, for which at least one of the methods had yielded in at least one subregion a mean cartilage thickness value that was more than five standard deviations above or below the average over all methods.^28^ If an outlier was detected, the entire knee joint was removed from subsequent analyses. For inter-method comparisons of mean cartilage thickness per (sub)region and computation times, repeated measures one-way analysis of variances (ANOVA) was performed for each (sub)region using Graph Pad Prism (v9.0, San Diego, CA).^29^ The Tukey-Kramer test was employed post hoc and multiplicity-adjusted p-values were computed to account for the multiple comparisons. The family-wise alpha threshold was set to 0.01 to reduce the number of statistically significant, but clinically (most likely) not relevant findings. Bland-Altman analysis was performed to further assess the magnitude of the pair-wise inter-method differences. In addition, Lin’s concordance correlation coefficient^30^ was calculated for all pair-wise comparisons using previously published Python code.^31^

## Results

3D-MN, 3D-NN, 3D-RT, 2D-CN, and 2D-SN produced 11 (2.1%), 13 (2.6%), 21 (4.1%), 16 (3.2%), and 10 (2.0%) non-overlapping outliers, respectively, that were discarded from further evaluation. In addition, the computation process was aborted in two knee joints (0.4%) for unknown reasons. Thus, 434 knee joints were included in the analysis.

**Figure 3.**
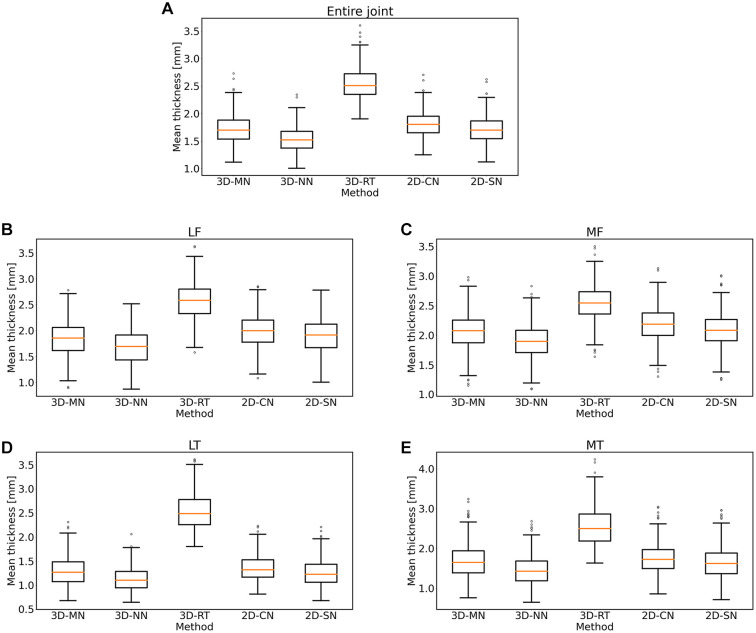
Box plots of the mean cartilage thickness values as a function of method. Shown are the mean thickness values for the entire knee joint (**A**), the lateral femoral cartilage (**B**), the medial femoral cartilage (**C**), the lateral tibial cartilage (**D**), and the medial tibial cartilage (**E**). Altogether, 434 knee joints were included, i.e., pre-identified outliers had been excluded. Orange lines indicate medians, the heights of the boxes indicate the interquartile ranges (IQRs), and the lengths of the whiskers indicate 1.5 times the IQR, respectively. 3D-MN: 3D mesh normals, 3D-NN: 3D nearest neighbors, 3D-RT: 3D ray tracing, 2D-CN: 2D centerline normals, 2D-SN: 2D surface normals; LF = lateral femur; MF = medial femur; LT = lateral tibia; MT = medial tibia.

**Figure 4.**
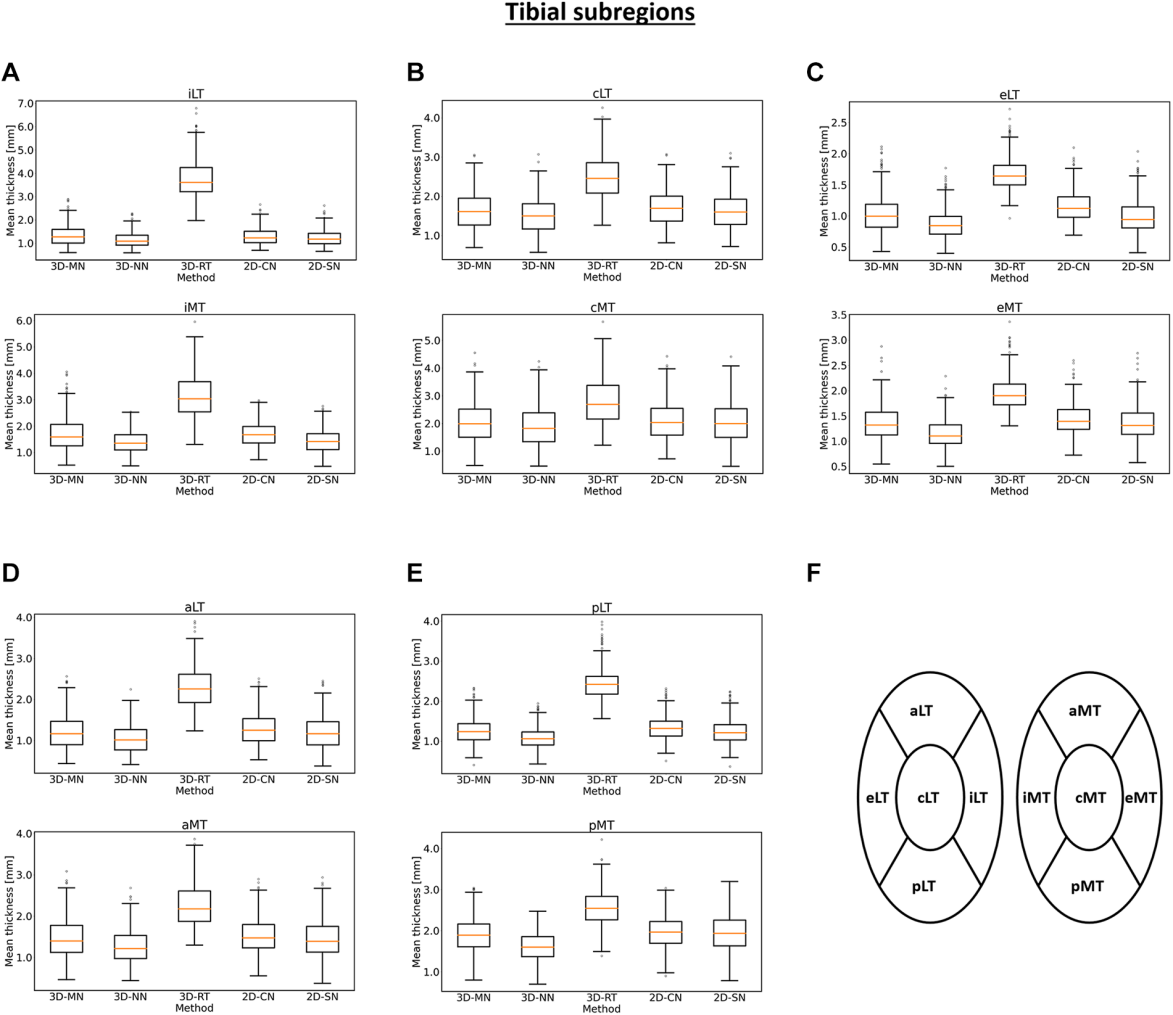
Box plots of the mean cartilage thickness values of the tibial subregions as a function of method. Box plots for the internal (**A**), central (**B**), external (**C**), anterior (**D**), and posterior (**E**) subregions of the tibia are shown as well as a schematic top view representation of their position in the joint (**F**). Acronyms as defined in **Table 1** and box plot organization as detailed in **Figure 3**. 3D-MN: 3D mesh normals, 3D-NN: 3D nearest neighbors, 3D-RT: 3D ray tracing, 2D-CN: 2D centerline normals, 2D-SN: 2D surface normals.

**Figure 5.**
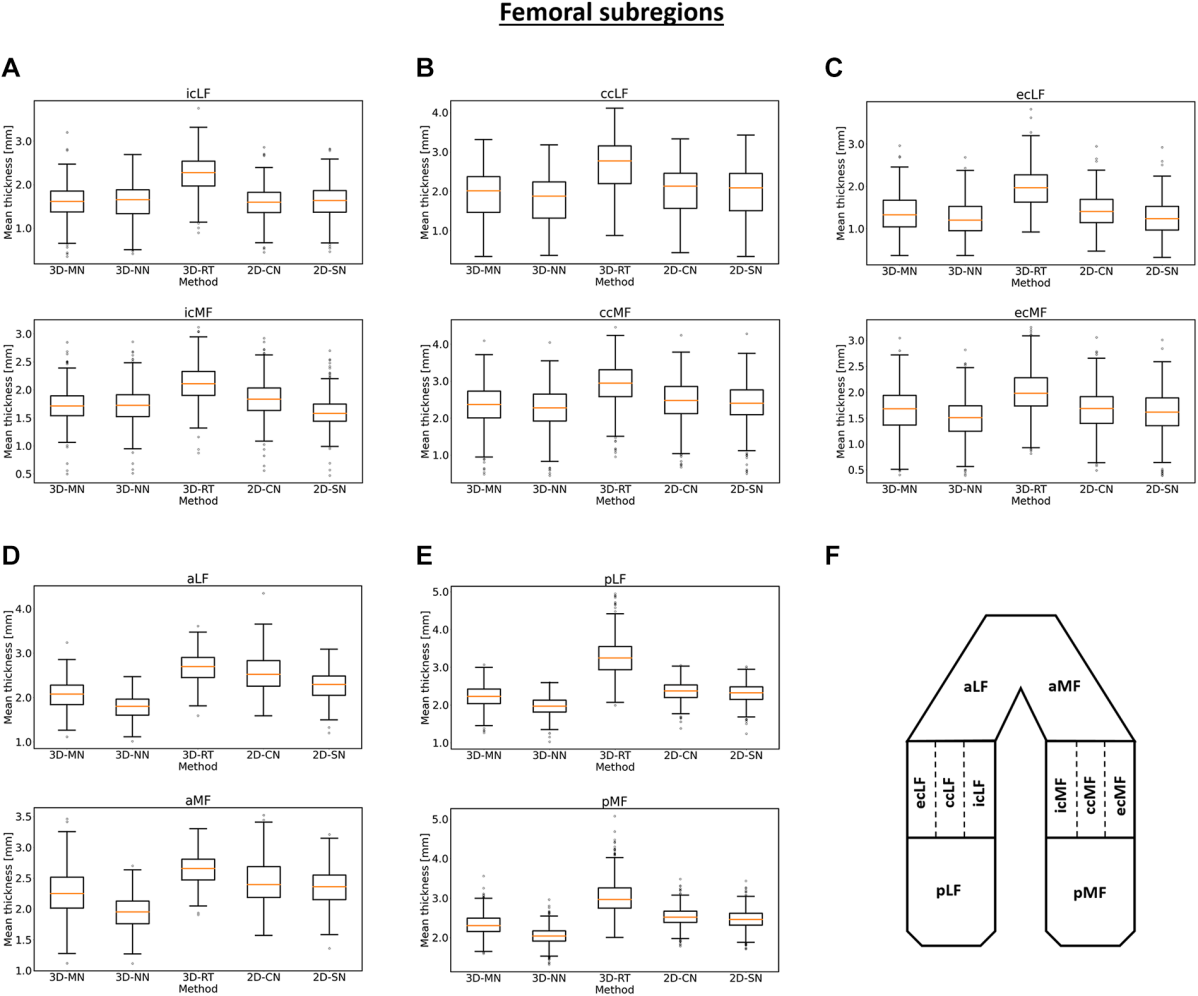
Box plots of the mean cartilage thickness values of the femoral subregions as a function of method. Box plots for the central (**A-C**), anterior (**D**), and posterior (**E**) subregions of the femur are shown as well as a schematic representation of their position in the joint (**F**). Acronyms as defined in **Table 1** and box plot organization as detailed in **Figure 3**. 3D-MN: 3D mesh normals, 3D-NN: 3D nearest neighbors, 3D-RT: 3D ray tracing, 2D-CN: 2D centerline normals, 2D-SN: 2D surface normals.

On a global and regional level, roughly similar mean cartilage thickness values were determined by all methods, except for 3D-RT (**Table 2**, **Fig. 3**). By trend, 3D-NN and 2D-CN tended to provide slightly lower and higher estimates than 3D-MN and 2D-SN. 3D-RT yielded highest mean cartilage thickness values. On a subregional level, these observations were largely confirmed, both for the tibia (**Fig. 4**) and the femur (**Fig. 5**). The 3D-RT delivered consistently larger mean cartilage thickness values throughout all tibial and femoral subregions.

**Table 2.** Mean Cartilage Thickness Values as a Function of Method and (Sub)Region.

	Cartilage Thickness (mm)					*P*-values
	3D-MN	3D-NN	3D-RT	2D-CN	2D-SN	
Global						
Entire joint	1.7 ± 0.3	1.5 ± 0.2	2.5 ± 0.3	1.8 ± 0.2	1.7 ± 0.2	*P* < 0.001
Regional						
Lateral tibia	1.3 ± 0.3	1.1 ± 0.3	2.5 ± 0.4	1.4 ± 0.3	1.3 ± 0.3	*P* < 0.001
Medial tibia	1.7 ± 0.4	1.5 ± 0.4	2.5 ± 0.5	1.7 ± 0.4	1.7 ± 0.4	*P* < 0.001
Lateral femur	1.8 ± 0.3	1.7 ± 0.3	2.6 ± 0.4	2.0 ± 0.3	1.9 ± 0.3	*P* < 0.001
Medial femur	2.1 ± 0.3	1.9 ± 0.3	2.5 ± 0.3	2.2 ± 0.3	2.1 ± 0.3	*P* < 0.001
Subregional (Tibia)						
cLT	1.6 ± 0.4	1.5 ± 0.4	2.5 ± 0.5	1.7 ± 0.4	1.6 ± 0.4	*P* < 0.001
iLT	1.3 ± 0.4	1.1 ± 0.4	3.8 ± 0.8	1.3 ± 0.3	1.2 ± 0.3	*P* < 0.001
eLT	1.0 ± 0.3	0.9 ± 0.3	1.7 ± 0.3	1.2 ± 0.2	1.0 ± 0.3	*P* < 0.001
aLT	1.2 ± 0.4	1.0 ± 0.4	2.3 ± 0.5	1.3 ± 0.4	1.2 ± 0.4	*P* < 0.001
pLT	1.3 ± 0.3	1.1 ± 0.3	2.4 ± 0.4	1.3 ± 0.3	1.2 ± 0.3	*P* < 0.001
cMT	2.0 ± 0.7	1.9 ± 0.7	2.8 ± 0.8	2.1 ± 0.7	2.1 ± 0.7	*P* < 0.001
iMT	1.7 ± 0.6	1.4 ± 0.6	3.2 ± 0.8	1.7 ± 0.4	1.4 ± 0.4	*P* < 0.001
eMT	1.4 ± 0.4	1.1 ± 0.3	2.0 ± 0.3	1.4 ± 0.3	1.4 ± 0.3	*P* < 0.001
aMT	1.5 ± 0.5	1.3 ± 0.5	2.3 ± 0.5	1.5 ± 0.4	1.5 ± 0.4	*P* < 0.001
pMT	1.9 ± 0.4	1.6 ± 0.4	2.6 ± 0.4	2.0 ± 0.4	1.9 ± 0.4	*P* < 0.001
Subregional (femur)						
ecLF	1.4 ± 0.5	1.2 ± 0.4	2.0 ± 0.5	1.4 ± 0.4	1.3 ± 0.4	*P* < 0.001
ccLF	1.9 ± 0.6	1.8 ± 0.6	2.7 ± 0.7	2.0 ± 0.6	2.0 ± 0.6	*P* < 0.001
icLF	1.6 ± 0.4	1.6 ± 0.4	2.2 ± 0.4	1.6 ± 0.4	1.6 ± 0.4	*P* < 0.001
aLF	2.1 ± 0.3	1.8 ± 0.3	2.7 ± 0.3	2.6 ± 0.4	2.3 ± 0.3	*P* < 0.001
pLF	2.2 ± 0.3	2.0 ± 0.3	3.3 ± 0.5	2.4 ± 0.3	2.3 ± 0.3	*P* < 0.001
ecMF	1.7 ± 0.4	1.5 ± 0.4	2.0 ± 0.4	1.7 ± 0.4	1.6 ± 0.4	*P* < 0.001
ccMF	2.3 ± 0.6	2.3 ± 0.6	2.9 ± 0.6	2.5 ± 0.6	2.4 ± 0.6	*P* < 0.001
icMF	1.7 ± 0.3	1.7 ± 0.3	2.1 ± 0.3	1.8 ± 0.3	1.6 ± 0.3	*P* < 0.001
aMF	2.3 ± 0.4	1.9 ± 0.4	2.6 ± 0.3	2.4 ± 0.4	2.4 ± 0.3	*P* < 0.001
pMF	2.3 ± 0.3	2.0 ±.03	3.0 ± 0.5	2.5 ± 0.3	2.4 ± 0.3	*P* < 0.001

Data are mean ± standard deviation [mm]. Inter-method comparisons were performed by repeated measures one-way ANOVA and respective p-values are given. Post-hoc test results are indicated in Supplementary Table S1. Acronyms and subregions as defined in **Table 1** and as visualized in **Figure 1**.

3D-MN = 3D mesh normals; 3D-NN = 3D nearest neighbors; 3D-RT = 3D ray tracing; 2D-CN = 2D centerline normals; 2D-SN = 2D surface normals.

Significant inter-method differences were found for all (sub)regions and the entire joint (Suppl. Table S1). Post-hoc testing revealed significance for nearly all pair-wise comparisons, except for some comparisons of 3D-MN vs. 2D-SN across the different (sub)regions and the internal central lateral femur (icLF) across the different pair-wise comparisons. Correspondingly, lowest mean absolute inter-method differences (indicating closest correspondence) were found between 3D-MN and 2D-SN (Suppl. Table S2), accompanied by highest Bland-Altman agreement, that is, lowest bias and smallest confidence intervals (Suppl. Table S3), and highest concordance correlation coefficients (CCCs; Suppl. Table S4). When 3D-RT was involved, mean absolute inter-method differences were as large as 2.47 mm (versus 2D-CN).

As far as the average number of individual thickness measurements per knee joint is concerned, 3D-MN, 3D-NN, 2D-CN, and 2D-SN were characterized by similar numbers of individual measurements, that is, 8,600 (for the tibia) and 17,000 to 18,000 (for the femur) (**Table 3**). In contrast, 3D-RT was based on less than half as many individual cartilage thickness measurements.

Average computation times per knee joint were 2.9 ± 1.0 s (2D-SN), 4.1 ± 1.2 s (3D-MN), 5.3 ± 0.6 s (3D-NN), 133 ± 29 s (2D-CN), and 351 ± 10 s (3D-RT). Significant differences were found for all pair-wise post-hoc comparisons (*P* ≤ 0.001).

**Table 3.** Average Number of Individual Thickness Measurements Performed in Tibia and Femur as a Function of Method.

Method	Average Number of Measurements (Tibia)	Average Number of Measurements (Femur)
3D-MN	(8.6 ± 1.4) × 10^3^	(1.8 ± 0.3) × 10^4^
3D-NN	(8.6 ± 1.4) × 10^3^	(1.8 ± 0.3) × 10^4^
3D-RT	(9.6 ± 1.0) × 10^2^	(7.4 ± 0.4) × 10^3^
2D-CN	(8.6 ± 1.4) × 10^3^	(1.7 ± 0.3) × 10^4^
2D-SN	(8.6 ± 1.4) × 10^3^	(1.7 ± 0.3) × 10^4^

Average number of measurements were evaluated on a subset of 50 segmented knee joints. Acronyms and subregions as defined in **Table 1** and as visualized in **Figure 1**.

3D-MN = 3D mesh normals; 3D-NN = 3D nearest neighbors; 3D-RT = 3D ray tracing; 2D-CN = 2D centerline normals; 2D-SN = 2D surface normals.

## Discussion

The most important findings of our study are that (1) automatic cartilage thickness measurements are largely affected by the underlying method of determination and that (2) the most efficient methods, that is, 2D-SN, 3D-MN, and 3D-NN, are also quantitatively closest to each other in a large subset of the OAI baseline cohort. Despite substantial research efforts over the past years and decades that focused on the development of pharmacologic^32,33^ and non-pharmacologic^34^ chondroprotective therapies, a comprehensive and systematic inter-method comparison of MRI-based cartilage thickness measurements is of clinical and scientific interest.

In our efforts to conduct such a comparison, we included methods that are well established in the field, i.e., 3D-MN^6,8,11,13^ and 3D-NN,^8-12^ as well as alternative methods that have not yet been applied to medical image analysis, that is, 3D-RT, 2D-CN, and 2D-SN. While such methods are established in the fields of engineering and mathematics, where ray tracing and polynomial function fitting have been studied for decades,^35,36^ their potential use in measuring cartilage thickness remains to be studied. Furthermore, given the nature of cross-sectional MR images, we also included three methods that work in the 3D space, that is, 3D-MN, 3D-NN, and 3D-RT, and two methods that treat the segmented data slice by slice, that is, 2D-CN and 2D-SN. For comprehensive analysis, tibial and femoral cartilage and their (sub)regions were included into our analysis. Also, all methods were chosen deliberately to be able to accommodate the tissue’s geometry, in particular at the femur, where the mean curvature is approximately 4.4 m^-1^, and the range is large at -20.0 m^-1^ to 27.2 m^-1^.^37^ In all, 3D-MN, 2D-CN, and 2D-SN made use of normal vectors for point-wise cartilage thickness measurements; 3D-NN identified the neighboring voxel at a minimum distance, which should approximate a surface normal for a sufficiently fine voxel grid; and 3D-RT placed spheres near the cartilage volume as origins of rays that were used for thickness estimation.

3D-MN and 2D-SN were quantitatively closest in terms of lowest absolute inter-method differences and least significant pair-wise differences. Mechanistically, **2D-SN** approximates the cartilage surfaces using polynomial functions that are -by design- unable to approximate higher frequency changes such as fibrillation and fissuring that are characteristic for incipient and early degeneration.^38^ Consequently, these signs of surface disintegration are likely smoothed out, which may challenge their identification. A potential improvement to the 2D-SN method would be to incorporate a B-spline approach, that is, to fit subsections of the cartilage outline piecewise with continuous transitions in between the fit functions.^39,40^ Yet, with regard to the limited in-plane resolution of clinical MRI sequences, in our study 0.36 x 0.36 mm², the evaluation of the earliest changes of the cartilage surface by MRI remains elusive. For example, the loss of the lamina splendens, which constitutes the most superficial cartilage layer, stabilizes the tissue mechanically, and has a mean thickness of approximately 6 to 15 μm,^41^ cannot be visualized anyway. In contrast, **3D-MN** does not rely on fitting of polynomial functions but uses meshes instead to delineate the surface and to define normals at the respective vertices. Because point-wise measurements (as normals to the lower tissue surface) are used, focal surface alterations (such as fissures) may be missed when mesh elements are too large. On the other hand, when mesh elements are too small, computational burden may be too high for time-efficient computation. In our implementation, the size of the mesh elements is predefined by the resolution of the voxel grid. When evaluating the computational burden, another aspect is important, too: For the implementation of the 3D-MN method, we could resort to pre-programmed algorithms that were available as optimized library functions, which made the method less computationally expensive and, thus, much faster.

As a modification of the 2D-SN method, **2D-CN** is expected to be more robust against non-ideal fitting outcomes: Even if the centerline of the 2D cartilage section is not perfectly met by the fit function, its normals will still cut the cartilage in a close-to-perpendicular manner with regard to the ideal centerline and provide accurate measurements. Numerically, 2D-CN yielded slightly higher mean cartilage thickness values than 2D-SN with mean absolute differences of 0.1 mm to 0.3 mm, that is, less than a voxel. In lack of a reference, the exact reason for this observation remains speculative, yet 2D-CN may catch for local thickness variations and surface protuberances (i.e., peaks) more efficiently that 2D-SN as the latter tends to smoothen out such surface irregularities altogether (i.e., peaks and valleys) by fitting a function that courses less superficially. As a result, statistically significant different were found between 2D-CN and 2D-SN in all subregions except for the icLF. Another important difference relates to computation times. While the intersection points between normals and the point cloud of cartilage surface voxels can only be determined iteratively for the 2D-CN method, that is, following each normal step-by-step until it intersects with a surface voxel, the intersection between normals and the surface outline can easily be calculated for the 2D-SN method, as both are represented by analytical functions. This renders 2D-SN much more computationally efficient (with an average processing time of less than 3 s per joint) than 2D-CN (133 s). Even though the search for the intersections may be -in principle- rendered more efficient,^42^ this aspect may be particularly relevant for large-scale analyses.

As a straightforward and plausible approach, **3D-NN** identifies the closest voxel on the opposite surface and, thus, computes the smallest point-to-point distances between the upper and lower tissue surfaces. In our study, 3D-NN was as time efficient as the 3D-MN and 2D-SN methods, while yielding the lowest thickness values of all methods under investigation. This observation is plausible as the 3D-NN-based prediction is conservative and commonly constitutes the lower bound of thickness estimations, which is well in line with earlier literature data that indicated lowest thickness values for 3D-NN.^11^ When data are noisy and, consequently, segmented outlines become less accurate, 3D-NN has been shown to yield even lower thickness values, even though the method is relatively robust in the presence of noise.^11^ It should be noted that 3D-NN suffers from high computational burden when implemented as a simple brute-force search. Modifications such as the KD-Tree search used in our study or local distance maps^43^ have alleviated this problem.

In contrast, **3D-RT** yielded the highest thickness values of all methods under investigation, thereby constituting the upper bound of thickness estimations, and, most likely, provided a substantial overestimation of the actual thickness. In consequence, CCCs of pair-wise comparisons involving 3D-RT were much lower (i.e., below 0.4) than for the other methods, and, similarly, low inter-method agreement as per Bland-Altman analysis was found. For the femur, the spheres were positioned near the focal point of the curved cartilage volume so that the rays penetrated the tissue at close-to-perpendicular angles. For the tibia, where the cartilage is relatively flat and “uncurved,” the method provided more overestimation than for the femur, which is plausible, too, as the rays did pass through the tibia at various angles (including the right angle) because the sphere was placed at a defined distance to the tissue. Consequently, the more the rays deviate from the perpendicular, the larger the overestimation of their thickness measurement will be because the rays simply travel longer distances in-between the two intersection points, that is, cartilage surfaces. The apparent overestimation of the femoral cartilage thickness suggests that a substantial number of rays fail to penetrate the cartilage surfaces perpendicularly, even in the curved and close-to-convexly shaped volumes. Moreover, the small number of thickness measurements (versus the large number of rays departing from eight spheres) suggest that many rays never penetrated the tissue which only occupied a small solid angle and would require further adaptive refinement of ray directions in the future. Consequently, the method was computationally very expensive and resulted in the longest processing times of all methods. These aspects preclude the method -as it is- from being applied clinically.

For future clinical studies and clinical practice, investigators should be aware of the substantial inter-method differences. Such differences may be larger than the average loss in cartilage height per year that depends on patient-, joint-, and tissue-related factors, yet is usually set at 0.1 mm over 2 years in untreated individuals (medial compartment).^32,44^

Our study has several limitations. First, no true gold standard for the cartilage thickness measurements was available which is why we only performed inter-method comparisons and cannot provide measures of accuracy. Second, we studied five select methods only, including two of the most frequently used methods, that is, 3D-MN and 3D-NN, yet our study is by no means exhaustive. Third, computation times (as surrogates of computational burden) can only be compared to a limited extent as 3D-MN had been optimized before (in terms of pre-programmed library functions), while 2D-CN and 3T-RT had not. Furthermore, parallelization of processing was not uniformly implemented between methods. Importantly, our study did not include any automatic cartilage segmentation but investigated the actual thickness determination based on manual reference segmentation. The time needed for manual segmentation is much longer than the time required for the subsequent cartilage thickness calculations. Consequently, future efforts should be focused on both, that is, streamlining automated segmentation methods such as deep learning-based approaches^45,46^ as well as subsequent post-processing approaches. Fourth, we applied our methods to one dataset based on one sequence, that is, DESS, and one set of image acquisition parameters only. Along similar lines, the dataset was heavily tilted toward more severe OA grades. Future studies need to investigate how other sequences and parameters, for example, using larger slice thickness than 0.7 mm, as well as healthier cohorts, affect the results.

In conclusion, our analysis of five methods for automatic cartilage thickness determination based on segmented femoral and tibial cartilage outlines of a large subset of the OAI baseline cohort indicates that quantification accuracy and computational burden are largely affected by the underlying method. Approaches based on mesh and surface normals as well as nearest neighbor searches were particularly promising with respect to computation times, while also being quantitatively close.

## Supplemental Material

sj-docx-1-car-10.1177_19476035221144744 – Supplemental material for Getting Cartilage Thickness Measurements Right: A Systematic Inter-Method Comparison Using MRI Data from the Osteoarthritis Initiative Click here for additional data file. Supplemental material, sj-docx-1-car-10.1177_19476035221144744 for Getting Cartilage Thickness Measurements Right: A Systematic Inter-Method Comparison Using MRI Data from the Osteoarthritis Initiative by Teresa Nolte, Simon Westfechtel, Justus Schock, Matthias Knobe, Torsten Pastor, Elisabeth Pfaehler, Christiane Kuhl, Daniel Truhn and Sven Nebelung in CARTILAGE

## Appendix

### Positioning of the Spheres for 3D-RT

Let P be the point cloud representing a segmented cartilage region or subregion. Let $c=(c_{x},c_{y},c_{z})$ be the location of the center of the sphere from where the rays originate. Depending on the region or subregion, c was determined as follows

For P = cMF, P = cLF, P = pMF and P = pLF,

$$c_{x}=\underset{x}{\min}\;P+(\underset{x}{\max}\;P-\underset{x}{\min}\;P)\cdot \frac{1}{4}$$

$$c_{y}=\underset{y}{\min}\;P+(\underset{y}{\max}\;P-\underset{y}{\min}\;P)\cdot \frac{1}{2}$$

$$c_{z}=\underset{z}{\min}\;P+(\underset{z}{\max}\;P-\underset{z}{\min}\;P)\cdot \frac{1}{2}$$

For P=aMF and P=aLF,

$$c_{x}=\underset{x}{\min}\;P+(\underset{x}{\max}\;P-\underset{x}{\min}\;P)\cdot \frac{3}{4}$$

$$c_{y}=\underset{y}{\min}\;P+(\underset{y}{\max}\;P-\underset{y}{\min}\;P)\cdot \frac{1}{2}$$

$$c_{z}=\underset{z}{\min}\;P+(\underset{z}{\max}\;P-\underset{z}{\min}\;P)\cdot \frac{1}{4}$$

For P=MT and P=LT,

$$c_{x}=\underset{x}{\min}\;P+(\underset{x}{\max}\;P-\underset{x}{\min}\;P)\cdot \frac{1}{2}$$

$$c_{y}=\underset{y}{\min}\;P+(\underset{y}{\max}\;P-\underset{y}{\min}\;P)\cdot \frac{1}{2}$$

$$c_{z}=\underset{z}{\max}\;P\cdot \frac{5}{4}$$
