# Supplementary material for: Getting Cartilage Thickness Measurements Right: A Systematic Inter-Method Comparison Using MRI Data from the Osteoarthritis Initiative
Source: Cartilage. 2023 Jan 19;14(1):26–38. doi: 10.1177/19476035221144744 (PMC10076900; doi:10.1177/19476035221144744)
Supplement: sj-docx-1-car-10.1177_19476035221144744 – Supplemental material for Getting Cartilage Thickness Measurements Right: A Systematic Inter-Method Comparison Using MRI Data from the Osteoarthritis Initiative [file sj-docx-1-car-10.1177_19476035221144744.docx]

## Supplementary Table 1

**Supplementary Table 1: Results of pair-wise post-hoc inter-method comparisons of mean cartilage thickness values.** Following repeated measures one-way ANOVA, the Tukey-Kramer post-hoc test was performed. Levels of statistical significance were stratified as “ns”, “*”, “**”, and “***” to indicate p>0.05, 0.01<p≤0.05, 0.001<p≤0.01, and p≤0.001^29^. Acronyms and subregions as defined in **Table 1** and as visualized in **Figure 1**.

|  | | Pair-wise Post-hoc Test | | | | | | | | | |
| --- | --- | --- | --- | --- | --- | --- | --- | --- | --- | --- | --- |
|  |  | 2D-CN vs. 3D-MN | 2D-CN vs. 3D-RT | 2D-CN vs. 2D-SN | 2D-CN vs. 3D-NN | 3D-MN vs. 3D-RT | 3D-MN vs. 2D-SN | 3D-MN vs. 3D-NN | 3D-RT vs. 2D-SN | 3D-RT vs. 3D-NN | 2D-SN vs. 3D-NN |
| Global | Entire joint | *** | *** | *** | *** | *** | ns | *** | *** | *** | *** |
| Regional | LT | *** | *** | *** | *** | *** | *** | *** | *** | *** | *** |
|  | MT | *** | *** | *** | *** | *** | *** | *** | *** | *** | *** |
|  | LF | *** | *** | *** | *** | *** | *** | *** | *** | *** | *** |
|  | MF | *** | *** | *** | *** | *** | *** | *** | *** | *** | *** |
| Subregional | cLT | *** | *** | *** | *** | *** | ns | *** | *** | *** | *** |
|  | iLT | *** | *** | *** | *** | *** | *** | *** | *** | *** | *** |
|  | eLT | *** | *** | *** | *** | *** | *** | *** | *** | *** | *** |
|  | aLT | *** | *** | *** | *** | *** | *** | *** | *** | *** | *** |
|  | pLT | *** | *** | *** | *** | *** | *** | *** | *** | *** | *** |
|  | cMT | *** | *** | *** | *** | *** | ns | *** | *** | *** | *** |
|  | iMT | ns | *** | *** | *** | *** | *** | *** | *** | *** | *** |
|  | eMT | *** | *** | *** | *** | *** | ns | *** | *** | *** | *** |
|  | aMT | *** | *** | *** | *** | *** | *** | *** | *** | *** | *** |
|  | pMT | *** | *** | *** | *** | *** | *** | *** | *** | *** | *** |
|  | ecLF | *** | *** | *** | *** | *** | *** | *** | *** | *** | *** |
|  | ccLF | *** | *** | *** | *** | *** | *** | *** | *** | *** | *** |
|  | icLF | ** | *** | ** | ns | *** | ns | ns | *** | *** | ns |
|  | aLF | *** | *** | *** | *** | *** | *** | *** | *** | *** | *** |
|  | pLF | *** | *** | *** | *** | *** | *** | *** | *** | *** | *** |
|  | ecMF | ns | *** | *** | *** | *** | *** | *** | *** | *** | *** |
|  | ccMF | *** | *** | *** | *** | *** | *** | *** | *** | *** | *** |
|  | icMF | *** | *** | *** | *** | *** | *** | ns | *** | *** | *** |
|  | aMF | *** | *** | *** | *** | *** | *** | *** | *** | *** | *** |
|  | pMF | *** | *** | *** | *** | *** | *** | *** | *** | *** | *** |

## Supplementary Table 2

**Supplementary Table 2:** **Mean absolute pair-wise differences in mean cartilage thickness.** The smallest absolute differences per (sub-)region are indicated in **bold type**. Acronyms and subregions as defined in **Table 1** and as visualized in **Figure 1**.

|  | | Pair-wise mean absolute differences [mm] | | | | | | | | | |
| --- | --- | --- | --- | --- | --- | --- | --- | --- | --- | --- | --- |
|  |  | 2D-CN vs. 3D-MN | 2D-CN vs. 3D-RT | 2D-CN vs. 2D-SN | 2D-CN vs. 3D-NN | 3D-MN vs. 3D-RT | 3D-MN vs. 2D-SN | 3D-MN vs. 3D-NN | 3D-RT vs. 2D-SN | 3D-RT vs. 3D-NN | 2D-SN vs. 3D-NN |
| Global | Entire joint | 0.10 | 0.73 | 0.10 | 0.28 | 0.83 | **0.002** | 0.18 | 0.83 | 1.01 | 0.18 |
| Regional | LT | 0.06 | 1.18 | 0.10 | 0.22 | 1.24 | **0.04** | 0.16 | 1.28 | 1.40 | 0.12 |
|  | MT | 0.05 | 0.80 | 0.09 | 0.29 | 0.85 | **0.04** | 0.23 | 0.90 | 1.09 | 0.19 |
|  | LF | 0.15 | 0.58 | 0.10 | 0.31 | 0.73 | **0.05** | 0.16 | 0.68 | 0.89 | 0.21 |
|  | MF | 0.12 | 0.37 | 0.10 | 0.29 | 0.49 | **0.02** | 0.17 | 0.46 | 0.65 | 0.19 |
| Sub-regional | cLT | 0.08 | 0.79 | 0.08 | 0.20 | 0.87 | **0.01** | 0.12 | 0.87 | 0.99 | 0.11 |
|  | iLT | **0.04** | 2.47 | 0.07 | 0.14 | 2.42 | 0.11 | 0.19 | 2.54 | 2.61 | 0.07 |
|  | eLT | 0.12 | 0.52 | 0.17 | 0.29 | 0.64 | **0.05** | 0.16 | 0.69 | 0.81 | 0.12 |
|  | aLT | 0.07 | 1.00 | 0.08 | 0.24 | 1.07 | **0.01** | 0.17 | 1.09 | 1.24 | 0.15 |
|  | pLT | 0.08 | 1.11 | 0.10 | 0.25 | 1.19 | **0.01** | 0.17 | 1.21 | 1.36 | 0.15 |
|  | cMT | 0.05 | 0.69 | 0.05 | 0.20 | 0.75 | **0.01** | 0.15 | 0.74 | 0.90 | 0.16 |
|  | iMT | **0.01** | 1.46 | 0.25 | 0.30 | 1.45 | 0.26 | 0.31 | 1.72 | 1.77 | 0.05 |
|  | eMT | 0.07 | 0.52 | 0.07 | 0.30 | 0.59 | **<0.01** | 0.23 | 0.59 | 0.82 | 0.23 |
|  | aMT | 0.06 | 0.74 | 0.07 | 0.26 | 0.80 | **0.01** | 0.20 | 0.81 | 0.99 | 0.19 |
|  | pMT | 0.08 | 0.60 | 0.03 | 0.37 | 0.68 | **0.06** | 0.29 | 0.62 | 0.97 | 0.35 |
|  | ecLF | 0.05 | 0.56 | 0.16 | 0.19 | 0.62 | 0.11 | 0.14 | 0.73 | 0.76 | **0.03** |
|  | ccLF | 0.11 | 0.64 | **0.03** | 0.23 | 0.74 | 0.08 | 0.12 | 0.67 | 0.86 | 0.20 |
|  | icLF | 0.02 | 0.66 | 0.02 | -0.02 | 0.64 | **<0.01** | **<0.01** | 0.64 | 0.64 | **<0.01** |
|  | aLF | 0.50 | 0.11 | 0.28 | 0.77 | 0.61 | **0.22** | 0.27 | 0.39 | 0.88 | 0.49 |
|  | pLF | 0.13 | 0.93 | **0.05** | 0.40 | 1.06 | 0.07 | 0.27 | 0.98 | 1.33 | 0.35 |
|  | ecMF | **<0.01** | 0.35 | 0.04 | 0.15 | 0.35 | 0.04 | 0.15 | 0.40 | 0.51 | 0.11 |
|  | ccMF | 0.12 | 0.46 | **0.06** | 0.20 | 0.58 | **0.06** | 0.08 | 0.52 | 0.66 | 0.14 |
|  | icMF | 0.11 | 0.28 | 0.24 | 0.11 | 0.40 | 0.12 | **<0.01** | 0.52 | 0.39 | 0.12 |
|  | aMF | 0.18 | 0.20 | **0.09** | 0.49 | 0.38 | **0.09** | 0.32 | 0.29 | 0.69 | 0.40 |
|  | pMF | 0.19 | 0.53 | **0.06** | 0.48 | 0.72 | 0.13 | 0.29 | 0.60 | 1.01 | 0.42 |

## Supplementary Table 3

**Supplementary Table 3: Results of Bland Altman Analysis.** Bias, lower (L.) 95% limit of agreement (LoA) and upper (U.) 95% LoA are indicated for every pair-wise comparison. The lowest absolute bias per (sub-)region is indicated in **bold type**. Acronyms and subregions as defined in **Table 1** and as visualized in **Figure 1**.

|  | | | Pair-wise Bland Altman results [mm] | | | | | | | | | |
| --- | --- | --- | --- | --- | --- | --- | --- | --- | --- | --- | --- | --- |
|  |  |  | 2D-CN vs. 3D-MN | 2D-CN vs. 3D-RT | 2D-CN vs. 2D-SN | 2D-CN vs. 3D-NN | 3D-MN vs. 3D-RT | 3D-MN vs. 2D-SN | 3D-MN vs. 3D-NN | 3D-RT vs. 2D-SN | 3D-RT vs. 3D-NN | 2D-SN vs. 3D-NN |
| Global | Entire joint | Bias  L. 95% LoA  U. 95% LoA | 0.10 | -0.73 | 0.10 | 0.28 | -0.83 | **0.002** | 0.18 | 0.83 | 0.10 | -0.73 |
|  |  |  | 0.01 | -0.91 | 0.04 | 0.19 | -1.00 | -0.07 | 0.07 | 0.65 | 0.01 | -0.91 |
|  |  |  | 0.18 | -0.56 | 0.15 | 0.36 | -0.66 | 0.08 | 0.30 | 1.01 | 0.18 | -0.56 |
| Regional | LT | Bias  L. 95% LoA  U. 95% LoA | 0.06 | -1.18 | 0.10 | 0.22 | -1.24 | **0.04** | 0.16 | 1.28 | 0.06 | -1.18 |
|  |  |  | -0.05 | -1.54 | 0.03 | 0.13 | -1.58 | -0.06 | 0.01 | 0.94 | -0.05 | -1.54 |
|  |  |  | 0.17 | -0.82 | 0.17 | 0.32 | -0.90 | 0.14 | 0.31 | 1.62 | 0.17 | -0.82 |
|  | MT | Bias  L. 95% LoA  U. 95% LoA | 0.05 | -0.80 | 0.09 | 0.29 | -0.85 | **0.04** | 0.23 | 0.90 | 0.05 | -0.80 |
|  |  |  | -0.10 | -1.15 | 0.02 | 0.18 | -1.15 | -0.09 | 0.05 | 0.55 | -0.10 | -1.15 |
|  |  |  | 0.21 | -0.45 | 0.17 | 0.40 | -0.56 | 0.17 | 0.42 | 1.24 | 0.21 | -0.45 |
|  | LF | Bias  L. 95% LoA  U. 95% LoA | 0.15 | -0.58 | 0.10 | 0.31 | -0.73 | **-0.05** | 0.16 | 0.68 | 0.15 | -0.58 |
|  |  |  | 0.01 | -0.87 | -0.02 | 0.15 | -1.03 | -0.16 | 0.01 | 0.40 | 0.01 | -0.87 |
|  |  |  | 0.30 | -0.29 | 0.23 | 0.48 | -0.43 | 0.05 | 0.31 | 0.96 | 0.30 | -0.29 |
|  | MF | Bias  L. 95% LoA  U. 95% LoA | 0.12 | -0.37 | 0.10 | 0.29 | -0.49 | **-0.02** | 0.17 | 0.46 | 0.12 | -0.37 |
|  |  |  | -0.02 | -0.59 | 0.00 | 0.15 | -0.74 | -0.14 | 0.03 | 0.25 | -0.02 | -0.59 |
|  |  |  | 0.26 | -0.14 | 0.20 | 0.43 | -0.24 | 0.09 | 0.31 | 0.67 | 0.26 | -0.14 |
| Sub-regional | cLT | Bias  L. 95% LoA  U. 95% LoA | 0.08 | -0.79 | 0.08 | 0.20 | -0.87 | **0.01** | 0.12 | 0.87 | 0.08 | -0.79 |
|  |  |  | -0.08 | -1.39 | -0.09 | 0.02 | -1.39 | -0.13 | -0.03 | 0.32 | -0.08 | -1.39 |
|  |  |  | 0.23 | -0.19 | 0.26 | 0.38 | -0.34 | 0.14 | 0.27 | 1.43 | 0.23 | -0.19 |
|  | iLT | Bias  L. 95% LoA  U. 95% LoA | -0.04 | -2.47 | **0.07** | 0.14 | -2.42 | 0.11 | 0.19 | 2.54 | -0.04 | -2.47 |
|  |  |  | -0.39 | -3.67 | -0.09 | -0.05 | -3.67 | -0.29 | -0.26 | 1.36 | -0.39 | -3.67 |
|  |  |  | 0.30 | -1.26 | 0.23 | 0.34 | -1.18 | 0.52 | 0.63 | 3.71 | 0.30 | -1.26 |
|  | eLT | Bias  L. 95% LoA  U. 95% LoA | 0.12 | -0.52 | 0.17 | 0.29 | -0.64 | **0.05** | 0.16 | 0.69 | 0.12 | -0.52 |
|  |  |  | -0.08 | -0.73 | -0.01 | 0.13 | -0.88 | -0.12 | -0.04 | 0.50 | -0.08 | -0.73 |
|  |  |  | 0.33 | -0.30 | 0.35 | 0.45 | -0.41 | 0.22 | 0.37 | 0.88 | 0.33 | -0.30 |
|  | aLT | Bias  L. 95% LoA  U. 95% LoA | 0.07 | -1.00 | 0.08 | 0.24 | -1.07 | **0.01** | 0.17 | 1.09 | 0.07 | -1.00 |
|  |  |  | -0.03 | -1.40 | -0.01 | 0.10 | -1.46 | -0.07 | -0.02 | 0.70 | -0.03 | -1.40 |
|  |  |  | 0.17 | -0.60 | 0.18 | 0.37 | -0.68 | 0.10 | 0.35 | 1.47 | 0.17 | -0.60 |
|  | pLT | Bias  L. 95% LoA  U. 95% LoA | 0.08 | -1.11 | 0.10 | 0.25 | -1.19 | **0.01** | 0.17 | 1.21 | 0.08 | -1.11 |
|  |  |  | -0.01 | -1.55 | 0.02 | 0.15 | -1.63 | -0.05 | 0.03 | 0.77 | -0.01 | -1.55 |
|  |  |  | 0.17 | -0.67 | 0.17 | 0.35 | -0.75 | 0.08 | 0.31 | 1.65 | 0.17 | -0.67 |
|  | cMT | Bias  L. 95% LoA  U. 95% LoA | 0.05 | -0.69 | 0.05 | 0.20 | -0.75 | **-0.01** | 0.15 | 0.74 | 0.05 | -0.69 |
|  |  |  | -0.08 | -1.10 | -0.15 | 0.03 | -1.14 | -0.16 | -0.01 | 0.29 | -0.08 | -1.10 |
|  |  |  | 0.19 | -0.28 | 0.24 | 0.37 | -0.36 | 0.14 | 0.31 | 1.19 | 0.19 | -0.28 |
|  | iMT | Bias  L. 95% LoA  U. 95% LoA | **-0.01** | -1.46 | 0.25 | 0.30 | -1.45 | 0.26 | 0.31 | 1.72 | **-0.01** | -1.46 |
|  |  |  | -0.52 | -2.46 | 0.07 | 0.07 | -2.39 | -0.30 | -0.31 | 0.69 | -0.52 | -2.46 |
|  |  |  | 0.50 | -0.47 | 0.43 | 0.53 | -0.51 | 0.83 | 0.93 | 2.74 | 0.50 | -0.47 |
|  | eMT | Bias  L. 95% LoA  U. 95% LoA | 0.07 | -0.52 | 0.07 | 0.30 | -0.59 | **0.00003** | 0.23 | 0.59 | 0.07 | -0.52 |
|  |  |  | -0.11 | -0.92 | -0.13 | 0.14 | -0.98 | -0.17 | 0.03 | 0.16 | -0.11 | -0.92 |
|  |  |  | 0.26 | -0.12 | 0.28 | 0.46 | -0.21 | 0.17 | 0.43 | 1.03 | 0.26 | -0.12 |
|  | aMT | Bias  L. 95% LoA  U. 95% LoA | 0.06 | -0.74 | 0.07 | 0.26 | -0.80 | **0.01** | 0.20 | 0.81 | 0.06 | -0.74 |
|  |  |  | -0.08 | -1.14 | -0.04 | 0.14 | -1.14 | -0.08 | 0.03 | 0.42 | -0.08 | -1.14 |
|  |  |  | 0.20 | -0.34 | 0.19 | 0.38 | -0.45 | 0.10 | 0.36 | 1.19 | 0.20 | -0.34 |
|  | pMT | Bias  L. 95% LoA  U. 95% LoA | 0.08 | -0.60 | **0.03** | 0.37 | -0.68 | -0.06 | 0.29 | 0.62 | 0.08 | -0.60 |
|  |  |  | -0.08 | -1.02 | -0.10 | 0.16 | -1.05 | -0.23 | 0.09 | 0.16 | -0.08 | -1.02 |
|  |  |  | 0.25 | -0.18 | 0.15 | 0.58 | -0.31 | 0.12 | 0.48 | 1.09 | 0.25 | -0.18 |
|  | ecLF | Bias  L. 95% LoA  U. 95% LoA | 0.05 | -0.56 | 0.16 | 0.19 | -0.62 | 0.11 | 0.14 | 0.73 | 0.05 | -0.56 |
|  |  |  | -0.15 | -1.00 | 0.01 | 0.01 | -1.08 | -0.09 | -0.09 | 0.26 | -0.15 | -1.00 |
|  |  |  | 0.26 | -0.12 | 0.32 | 0.38 | -0.15 | 0.31 | 0.37 | 1.19 | 0.26 | -0.12 |
|  | ccLF | Bias  L. 95% LoA  U. 95% LoA | 0.11 | -0.64 | **0.03** | 0.23 | -0.74 | -0.08 | 0.12 | 0.67 | 0.11 | -0.64 |
|  |  |  | -0.03 | -1.10 | -0.15 | 0.02 | -1.19 | -0.22 | -0.04 | 0.20 | -0.03 | -1.10 |
|  |  |  | 0.24 | -0.18 | 0.21 | 0.43 | -0.30 | 0.07 | 0.29 | 1.13 | 0.24 | -0.18 |
|  | icLF | Bias  L. 95% LoA  U. 95% LoA | -0.02 | -0.66 | -0.02 | -0.02 | -0.64 | 0.00 | **-0.0005** | 0.64 | -0.02 | -0.66 |
|  |  |  | -0.20 | -0.98 | -0.25 | -0.31 | -1.00 | -0.26 | -0.34 | 0.34 | -0.20 | -0.98 |
|  |  |  | 0.17 | -0.33 | 0.21 | 0.28 | -0.28 | 0.26 | 0.34 | 0.93 | 0.17 | -0.33 |
|  | aLF | Bias  L. 95% LoA  U. 95% LoA | 0.50 | **-0.11** | 0.28 | 0.77 | -0.61 | -0.22 | 0.27 | 0.39 | 0.50 | **-0.11** |
|  |  |  | -0.09 | -0.74 | -0.28 | 0.16 | -0.94 | -0.44 | 0.05 | 0.16 | -0.09 | -0.74 |
|  |  |  | 1.08 | 0.51 | 0.83 | 1.37 | -0.28 | 0.01 | 0.50 | 0.62 | 1.08 | 0.51 |
|  | pLF | Bias  L. 95% LoA  U. 95% LoA | 0.13 | -0.93 | **0.05** | 0.40 | -1.05 | -0.07 | 0.27 | 0.98 | 0.13 | -0.93 |
|  |  |  | -0.10 | -1.77 | 0.02 | 0.29 | -1.92 | -0.31 | 0.04 | 0.13 | -0.10 | -1.77 |
|  |  |  | 0.36 | -0.09 | 0.09 | 0.51 | -0.19 | 0.16 | 0.51 | 1.82 | 0.36 | -0.09 |
|  | ecMF | Bias  L. 95% LoA  U. 95% LoA | **-0.00005** | -0.35 | 0.04 | 0.15 | -0.35 | 0.04 | 0.15 | 0.40 | **-0.00005** | -0.35 |
|  |  |  | -0.26 | -0.70 | -0.17 | -0.05 | -0.78 | -0.22 | -0.14 | 0.03 | -0.26 | -0.70 |
|  |  |  | 0.26 | -0.005 | 0.26 | 0.36 | 0.07 | 0.31 | 0.45 | 0.76 | 0.26 | -0.005 |
|  | ccMF | Bias  L. 95% LoA  U. 95% LoA | 0.12 | -0.46 | 0.06 | 0.20 | -0.58 | **-0.06** | 0.08 | 0.52 | 0.12 | -0.46 |
|  |  |  | -0.04 | -0.82 | -0.13 | 0.01 | -0.96 | -0.21 | -0.08 | 0.12 | -0.04 | -0.82 |
|  |  |  | 0.28 | -0.10 | 0.26 | 0.39 | -0.20 | 0.10 | 0.24 | 0.93 | 0.28 | -0.10 |
|  | icMF | Bias  L. 95% LoA  U. 95% LoA | 0.11 | -0.28 | 0.24 | 0.11 | -0.40 | 0.12 | **-0.003** | 0.52 | 0.11 | -0.28 |
|  |  |  | -0.10 | -0.61 | 0.00 | 0.01 | -0.78 | -0.03 | -0.21 | 0.11 | -0.10 | -0.61 |
|  |  |  | 0.33 | 0.05 | 0.47 | 0.21 | -0.02 | 0.27 | 0.21 | 0.93 | 0.33 | 0.05 |
|  | aMF | Bias  L. 95% LoA  U. 95% LoA | 0.18 | -0.20 | 0.09 | 0.49 | -0.38 | **-0.09** | 0.32 | 0.29 | 0.18 | -0.20 |
|  |  |  | -0.28 | -0.76 | -0.37 | 0.03 | -0.74 | -0.35 | 0.05 | 0.07 | -0.28 | -0.76 |
|  |  |  | 0.63 | 0.36 | 0.55 | 0.96 | -0.02 | 0.17 | 0.59 | 0.50 | 0.63 | 0.36 |
|  | pMF | Bias  L. 95% LoA  U. 95% LoA | 0.19 | -0.53 | **0.06** | 0.48 | -0.72 | -0.13 | 0.29 | 0.60 | 0.19 | -0.53 |
|  |  |  | -0.07 | -1.31 | 0.03 | 0.37 | -1.63 | -0.39 | 0.00 | -0.19 | -0.07 | -1.31 |
|  |  |  | 0.45 | 0.25 | 0.10 | 0.60 | 0.19 | 0.14 | 0.59 | 1.38 | 0.45 | 0.25 |

## Supplementary Table 4

**Supplementary Table 4: Lin’s Concordance Correlation Coefficients (CCC).** CCC was calculated pair-wise between the methods. The highest CCC per (sub-)region is indicated in **bold type**. Acronyms and subregions as defined in **Table 1** and as visualized in **Figure 1**.

|  | | Pair-wise CCC | | | | | | | | | |
| --- | --- | --- | --- | --- | --- | --- | --- | --- | --- | --- | --- |
|  |  | 2D-CN vs. 3D-MN | 2D-CN vs. 3D-RT | 2D-CN vs. 2D-SN | 2D-CN vs. 3D-NN | 3D-MN vs. 3D-RT | 3D-MN vs. 2D-SN | 3D-MN vs. 3D-NN | 3D-RT vs. 2D-SN | 3D-RT vs. 3D-NN | 2D-SN vs. 3D-NN |
| Global | Entire joint | 0.918 | 0.190 | 0.918 | 0.577 | 0.167 | **0.989** | 0.765 | 0.156 | 0.104 | 0.770 |
| Regional | LT | 0.955 | 0.107 | 0.926 | 0.710 | 0.108 | **0.974** | 0.813 | 0.098 | 0.076 | 0.888 |
|  | MT | 0.973 | 0.334 | 0.966 | 0.760 | 0.343 | **0.982** | 0.828 | 0.299 | 0.215 | 0.875 |
|  | LF | 0.881 | 0.368 | 0.936 | 0.656 | 0.280 | **0.976** | 0.870 | 0.306 | 0.197 | 0.815 |
|  | MF | 0.897 | 0.528 | 0.930 | 0.650 | 0.401 | **0.978** | 0.840 | 0.422 | 0.272 | 0.817 |
| Subregional | cLT | 0.969 | 0.344 | 0.961 | 0.882 | 0.336 | **0.988** | 0.948 | 0.322 | 0.280 | 0.945 |
|  | iLT | 0.892 | 0.052 | **0.951** | 0.874 | 0.058 | 0.814 | 0.726 | 0.050 | 0.050 | 0.951 |
|  | eLT | 0.845 | 0.298 | 0.775 | 0.547 | 0.258 | **0.943** | 0.781 | 0.217 | 0.148 | 0.877 |
|  | aLT | 0.977 | 0.251 | 0.971 | 0.810 | 0.243 | **0.994** | 0.884 | 0.236 | 0.168 | 0.899 |
|  | pLT | 0.954 | 0.125 | 0.942 | 0.690 | 0.119 | **0.993** | 0.823 | 0.113 | 0.080 | 0.847 |
|  | cMT | 0.992 | 0.666 | 0.988 | 0.951 | 0.648 | **0.994** | 0.972 | 0.646 | 0.566 | 0.967 |
|  | iMT | 0.887 | 0.199 | 0.835 | 0.771 | 0.260 | 0.764 | 0.700 | 0.147 | 0.149 | **0.960** |
|  | eMT | 0.932 | 0.336 | 0.920 | 0.613 | 0.325 | **0.966** | 0.743 | 0.296 | 0.172 | 0.737 |
|  | aMT | 0.976 | 0.401 | 0.977 | 0.817 | 0.404 | **0.995** | 0.886 | 0.383 | 0.274 | 0.895 |
|  | pMT | 0.958 | 0.415 | **0.986** | 0.642 | 0.385 | 0.969 | 0.756 | 0.413 | 0.212 | 0.681 |
|  | ecLF | 0.960 | 0.474 | 0.902 | 0.865 | 0.461 | 0.941 | 0.912 | 0.365 | 0.326 | **0.977** |
|  | ccLF | 0.981 | 0.638 | **0.989** | 0.926 | 0.580 | 0.986 | 0.973 | 0.625 | 0.502 | 0.940 |
|  | icLF | **0.969** | 0.391 | 0.953 | 0.924 | 0.411 | 0.944 | 0.910 | 0.416 | 0.414 | 0.959 |
|  | aLF | 0.372 | 0.629 | 0.574 | 0.191 | 0.306 | **0.759** | 0.642 | 0.534 | 0.161 | 0.392 |
|  | pLF | 0.824 | 0.119 | **0.978** | 0.437 | 0.101 | 0.879 | 0.606 | 0.109 | 0.070 | 0.509 |
|  | ecMF | 0.949 | 0.666 | **0.958** | 0.895 | 0.656 | 0.945 | 0.870 | 0.633 | 0.484 | 0.928 |
|  | ccMF | 0.969 | 0.723 | 0.979 | 0.931 | 0.629 | **0.986** | 0.981 | 0.663 | 0.578 | 0.951 |
|  | icMF | 0.884 | 0.640 | 0.709 | 0.932 | 0.466 | 0.890 | **0.941** | 0.326 | 0.507 | 0.845 |
|  | aMF | 0.704 | 0.485 | 0.733 | 0.337 | 0.468 | **0.887** | 0.606 | 0.608 | 0.211 | 0.497 |
|  | pMF | 0.714 | 0.215 | **0.968** | 0.336 | 0.102 | 0.802 | 0.529 | 0.190 | 0.094 | 0.406 |

**References:**

^29^ "Graphpad.com," [Online]. Available: https://www.graphpad.com/support/faq/what-is-the-meaning-of--or--or--in-reports-of-statistical-significance-from-prism-or-instat/. [Accessed 25 May 2022].
